# Outcomes of a Hybrid Ophthalmology Telemedicine Model for Outpatient Eye Care During COVID-19

**DOI:** 10.1001/jamanetworkopen.2022.26292

**Published:** 2022-08-25

**Authors:** Nedda Sanayei, Melanie M. Albrecht, Diana C. Martin, Nicolas Marin, Shaunt Fereshetian, Steven Baker, Manju L. Subramanian, Steven Ness, Nicole H. Siegel, Xuejing Chen

**Affiliations:** 1 Department of Ophthalmology, Boston Medical Center, Boston, Massachusetts; 2 Northeastern University, Boston, Massachusetts; 3 Boston University School of Medicine, Boston, Massachusetts; 4 Department of Ophthalmology, Boston University School of Medicine, Boston, Massachusetts

## Abstract

**Importance:**

The hybrid ophthalmology telemedicine model asynchronously pairs an imaging appointment by a technician with a subsequent virtual appointment by a clinician. Although it has been mentioned in several studies as an alternative to standard in-person care during the COVID-19 pandemic, outcomes of this alternative clinical care model remain to be evaluated.

**Objective:**

To investigate the outcomes associated with the hybrid ophthalmology telemedicine model during the COVID-19 pandemic for nonurgent and nonprocedural ophthalmological care.

**Design, Setting, and Participants:**

Retrospective, cross-sectional study of all hybrid visits scheduled during the year 2020 in a single academic, hospital-based eye clinic in Boston, Massachusetts. All hybrid ophthalmology telemedicine visits completed in the year 2020 by opthalmologists and optometrists were included. Data were analyzed from January to December 2020.

**Exposures:**

Hybrid telemedicine clinical encounters.

**Main Outcomes and Measures:**

Four outcome metrics were calculated: (1) need for subsequent procedure visit, (2) medication change, (3) nonurgent, and (4) urgent consultation with another eye clinician. Adverse outcomes were defined as irreversible vision loss and the need for additional in-person evaluation to reach a management decision.

**Results:**

From April 9 to December 30, 2020, 889 patients (506 female patients [56.9%]; mean [SD] age, 62.1 [14.5] years; age range, 13-98 years) completed 940 hybrid visits. The most common visit indications were glaucoma (424 visits [45.1%]) and retinal diseases (499 visits [53.1%]). A total of 25 visits (2.7%) led to a procedure, 22 visits (2.3%) led to a change in medication, and 44 visits (4.7%) were referred for nonurgent consultation with another subspecialty with no instances of urgent referrals. Sixteen patients (1.7%) were referred to the on-call clinician for a same-day emergency in-person visit or recommended for a subsequent standard in-person visit to reach a management decision. There were no cases of irreversible vision loss following a hybrid visit.

**Conclusions and Relevance:**

These findings suggest that with the appropriate patient selection and clinical setting, the hybrid ophthalmology telemedicine model may be a good alternative to standard in-person visits, particularly for patients with glaucoma and retinal diseases.

## Key Points

**Question:**

What are the outcomes associated with a hybrid ophthalmology telemedicine model, comprising an imaging appointment with a technician followed by a virtual appointment with a clinician, for patients with stable, nonprocedural eye concerns?

**Findings:**

In this cross-sectional study involving 889 patients, no cases of irreversible vision loss were seen, and only 1.7% of patients required a subsequent in-person evaluation to clarify management. Hybrid visits were primarily for glaucoma (45.1%) and retinal issues (53.1%).

**Meaning:**

These findings suggest that with the appropriate patient selection, the hybrid ophthalmology telemedicine model can be a good alternative to standard in-person visits, particularly for patients with glaucoma or retinal diseases.

## Teaser

This cross-sectional study evaluates outcomes of a hybrid ophthalmology telemedicine model for outpatient eye care during COVID-19.

## Introduction

Telemedicine is health care provided remotely using telecommunication tools such as telephones, smartphone devices, and computers with or without a video connection.^1,2^ In ophthalmology, telemedicine use has traditionally focused on a store-and-forward model for diabetic retinopathy, retinopathy of prematurity, macular degeneration, and glaucoma to bridge a lack of access due to distance or workforce limitations.^3,4^ These asynchronous telemedicine models involve acquiring images of the retina or optic nerve through a secure platform that sends the data to a remote eye care professional. Later, the eye care professional interprets the data and returns a management recommendation to the patient or image acquisition site. With the COVID-19 pandemic, telemedicine quickly assumed a new purpose: providing clinical care while limiting physical contact to reduce the transmission of a dangerous respiratory illness.

Although real-time video-based and telephone-based platforms have emerged as the primary models of ophthalmic telemedicine during the pandemic, these platforms are constrained by the limited, or complete lack of, ability to obtain objective data such as ocular vitals and physical examination elements. This is a particular hindrance in ophthalmic care, which traditionally has a high general reliance on objective data such as ocular vitals and the ophthalmic examination. The hybrid ophthalmology telemedicine model, comprising an in-person, nonmydriatic imaging appointment with a trained ophthalmic technician followed by a virtual appointment with the clinician, is an alternative model that can combine limited objective data with the benefits of telemedicine (see eTable 1 in the Supplement). The use of this model is particularly suited to providing nonurgent, nonprocedural, routine clinical care to a population of patients whose visits may otherwise have been postponed during the pandemic.

Currently, we are unaware of large-scale studies that have evaluated the outcomes of the hybrid ophthalmology telemedicine model despite multiple studies^4,5,6,7,8,9,10^ describing the model for eye care. Although the concept of a visit model that can emulate standard in-person visits for eye care with reduced physical contact sounds appealing, particularly during a global pandemic, the ability to deliver quality patient care cannot be sacrificed. This study reports on the outcomes of the hybrid ophthalmology telemedicine model implemented on a wide scale with multiple ophthalmic subspecialties and optometry in a hospital-based eye clinic in Boston, Massachusetts, during the COVID-19 pandemic. As the pandemic continues to evolve at the time of manuscript writing, lessons from our study may apply to future pandemics or even be incorporated into existing standards of care models.

## Methods

### Hybrid Ophthalmology Telemedicine Model

This is a retrospective cross-sectional study of all patients scheduled for a hybrid visit in the Department of Ophthalmology at Boston Medical Center in the year 2020. The Boston University institutional review board approval was obtained and the need for patient consent was waived in accordance with 45 CFR §46. The study followed the Strengthening the Reporting of Observational Studies in Epidemiology (STROBE) reporting guideline for cross-sectional studies.^11^

Patients for hybrid visits were either selected by the clinician through manual medical record review or direct scheduling following any visit type. In the early stages of the model when it was clear that the continued postponing of nonurgent visits was delaying care, clinicians reviewed the medical records of all encounters that were previously scheduled as a standard in-person visit and manually marked these visits as transition to hybrid visit, transition to virtual-only visit, keep as in-person visit, or postpone. The selection of patients was up to the discretion of the clinician, but clinicians were recommended to select stable, nonprocedural, non–vision-threatening diseases that could be evaluated with a standard protocol-based set of imaging and testing. As time went on and the population of nonreviewed, previously scheduled in-person visits decreased, clinicians and patients had more opportunities to decide together whether they wanted a follow-up visit to be in-person, virtual-only, or hybrid.

A clinic scheduler would then make both imaging and virtual appointments simultaneously and was given scripts to describe this new visit type to patients, emphasizing that adherence to both appointments constituted a single visit. To ensure that the ocular vitals and testing obtained at the imaging appointment were representative of a patient’s state at the time of the virtual appointment, all virtual appointments were scheduled within 14 days of the imaging appointment. To minimize exposures, a single technician obtained both ocular vitals and testing for each patient. If a patient missed their imaging appointment, a protocol was in place to automatically reschedule the imaging and virtual appointments. If a patient missed their virtual appointment, this appointment would be rescheduled. Additionally, if a patient missed their virtual appointment and their imaging appointment had suggested a progression of their disease requiring a management change, a certified letter was mailed to the patient’s home alerting them to this and prompting the patient to call the eye clinic.

During the imaging appointment, the technician collected basic ophthalmic vitals such as visual acuity, intraocular pressure, and a protocol-driven array of imaging and testing (eg, fundus photography, ocular coherence tomography, visual fields) according to the chief concern (eTable 2 in the Supplement). Technicians were also given a clear list of parameters on when to escalate care to the on-call ophthalmologist, such as severely elevated intraocular pressure or severe pain.

### Statistical Analysis

Electronic medical records of all patient encounters in the year 2020 were reviewed. All patient encounters were then categorized into hybrid, virtual-only, and standard in-person visits. Virtual-only visits did not have an in-person component and were telephone or video-based depending on patient and clinician preference. Interpreter services were used for non–English-speaking patients.

Patient-level data for all hybrid visits were automatically exported from the electronic medical record, including sex, age, race and ethnicity, and primary language. Race and ethnicity data were self-reported, limited by the options within the electronic medical records, and collected to evaluate equity in access to care. They were categorized into mutually exclusive groups: Asian, Black, Hispanic/Latino, non-Hispanic White, other, and declined. The primary language was also self-reported and further categorized as English and non-English.

Visit-level data extracted included the chief concern, all dates associated with the visit, the responsible ophthalmology subspecialty or optometry, and visit outcomes. This data was manually extracted by 6 reviewers (N.S. [ophthalmology resident], M.M.A. [undergraduate research assistant], D.C.M. [ophthalmic technician], N.M. [medical student], S.F. [medical student], and S.B. [undergraduate research assistant]) all trained by 2 reviewers (N.S. and X.C. [attending ophthalmologists]). In the rare situation of ambiguity in the medical records, the senior author (X.C.) made the final decision.

A hybrid visit was considered a no-show visit if the patients did not present for either the imaging or virtual appointment. Once an appointment was categorized as a no-show, any nonadherence of a subsequent rescheduled appointment was not included in no-show calculations. The hybrid no-show rate was compared with the overall clinical no-show rate during the same period, which was calculated by taking the number of all incomplete visit encounters, including hybrid and standard in-person visits, in the eye clinic divided by the total scheduled visit encounters in the eye clinic. For all completed hybrid visits, the number of days between imaging and virtual appointments was determined, and the percentage of visits that met our protocol goal of 14 days was calculated.

As with standard in-person visits and virtual-only telemedicine visits, a hybrid visit is positioned to provide an assessment and plan for the patient. We defined and analyzed the following 4 outcome measures according to situations where the management went beyond verbal counseling: (1) procedure visit: clinician requesting a return to clinic for a procedure (eg, intravitreal injection for new center-involving macular edema); (2) medication adjustment: a change in medications (eg, glaucoma medication titration); (3) nonurgent referral: referral to a different clinician, typically in another subspecialty, for additional evaluation in a nonurgent time frame; (4) urgent referral: referral to a different clinician, typically in another subspecialty, for additional evaluation within 1 month.

Adverse outcomes included irreversible vision loss due to a hybrid visit missing a worsening disease process or a new diagnosis. This was accessed at the clinical visit following the hybrid visit. We also evaluated the need for an extraneous in-person evaluation to reach a management plan because of limitations from the hybrid visit. All statistics were compiled and tabulated using Excel for Mac version 16.54 (Microsoft). Data were analyzed from January to December 2020.

## Results

A total of 940 hybrid visits associated with 889 patients (506 female patients [56.9%]; mean [SD] age, 62.1 [14.5] years; age range, 13-98 years) were completed in the year 2020, occurring from April 9 to December 30, 2020. For comparison, 6087 standard in-person visits occurred during the same period. A total of 424 patients (47.7%) self-identified as Black and 222(25.0%) as Hispanic/Latino; 343 (38.6%) self-reported a non-English primary language with Spanish, Haitian Creole, and Cape Verdean Creole as the predominant languages. Additional patient-level demographics are outlined in Table 1.

**Table 1.** Patient Demographics for All Completed Hybrid Visits in the Year 2020

Characteristics	Patients, No. (%) (N = 889)
Sex	
Male	383 (43.1)
Female	506 (56.9)
Age, y	
10-20	8 (0.9)
21-40	67 (7.5)
41-60	284 (31.9)
61-80	462 (52.0)
81-100	68 (7.6)
Race and ethnicity	
Asian	36 (4.0)
Black	424 (47.7)
Hispanic-Latino	222 (25.0)
Non-Hispanic White	101 (11.4)
Other^a^	11 (1.2)
Declined	95 (10.7)
Language	
English	546 (61.4)
Spanish	172 (19.3)
Haitian Creole	75 (8.4)
Cape Verdean Creole	23 (2.6)
Portuguese	20 (2.2)
Vietnamese	14 (1.6)
Other	39 (4.4)

^a^ Other includes the following: Native Hawaiian or Pacific Islander (3 participants), Native American (2 participants), Middle Eastern (2 participants), mixed race (Black and Asian [1 participant]), and other (not otherwise specified [3 participants]).

### Visit Adherence

Hybrid visits were fully completed 86.2% (940 visits) of the time. In 1.3% (14 visits) of all scheduled hybrid visits (1091 visits), the patient did not present for their imaging appointment. Of the patients who did present for imaging appointments, 94 (8.7 %) did not present for their virtual appointments. Only 43 patients (3.9%) missed both imaging and virtual appointments. Patients who rescheduled either imaging or virtual appointments before the appointment were not considered to have missed those appointments. The overall hybrid no-show rate was 13.8% (151 visits) which was lower than the 22.9% (monthly range, 18.5%-31.3%) no-show rate for all ophthalmologic visits from April to December 2020. Additionally, 49 patients (5.5%) completed more than 1 hybrid visit, with 1 patient completing 3 visits in 2020.

Of all completed hybrid visits, the virtual appointments occurred a mean (SD) of 11.1 (11.9) days (range, 0-132 days) after the imaging appointment with most virtual appointments taking place in 1 day (112 visits [11.9%]), 7 days (125 visits [13.3%]), and 14 days (44 visits [4.7%]) (Figure). In total, 728 patients with hybrid visits (77.4%) had their virtual appointment occur within our protocol goal of 14 days after the imaging appointment. For the year 2020, all virtual appointments were telephone-based.

**Figure.**
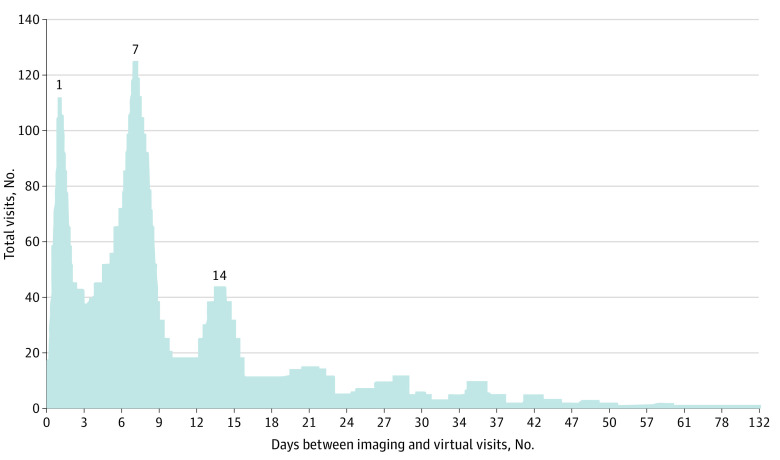
Time Between Virtual and Imaging Appointments for All Completed Hybrid Visits Figure excludes the 4 patients who were converted to same-day emergency in-person visits with the on-call team.

### Outcomes

The chief concern for the visits were primarily glaucoma (424 visits [45.1%]) and retinal diseases (499 visits [53.1%]). The remaining 1.8% (17 visits) spanned the realm of oculoplastics, neuro-ophthalmology, and cornea and refractive issues. Table 2 outlines the distribution of the primary visit diagnoses in detail.

**Table 2.** Primary Visit Diagnosis for All Completed Hybrid Visits in the Year 2020

Chief concern	Visits, No. (%) (N = 940)
Glaucoma	
Any	424 (45.1)
Suspect	232 (24.7)
Primary open angle	137 (14.6)
Low tension	24 (2.6)
Mixed mechanism	11 (1.2)
Other^a^	20 (2.1)
Retina	
Any	499 (53.1)
Nonproliferative diabetic retinopathy	130 (13.8)
Proliferative diabetic retinopathy	64 (6.8)
Retinal vein occlusion	32 (3.4)
With macular edema, not requiring recent treatment	18 (1.9)
Without macular edema	14 (1.4)
Age-related macular degeneration	30 (3.2)
Dry, no prior treatment	19 (2.0)
Wet, with scarring not requiring treatment	11 (1.2)
Diabetic retinopathy screening without known retinopathy	25 (2.7)
Epiretinal membrane	20 (2.1)
Plaquenil screening	20 (2.1)
History of rhegmatogenous retinal detachment repair	18 (1.9)
Retinal hole, break, or tear	18 (1.9)
Treated with laser retinopexy	14 (1.5)
Stable, not requiring treatment	4 (0.4)
Central serous chorioretinopathy	17 (1.8)
Miscellaneous inactive choroidal neovascular membrane^b^	12 (1.3)
Lamellar macular hole	11 (1.2)
Other retina^c^	102 (10.9)
Miscellaneous^d^	17 (1.8)

^a^ Other glaucoma includes chronic angle closure glaucoma (5 patients), pseudoexfoliative glaucoma (3 patients), anatomical narrow angle (2 patients), angle recession glaucoma (2 patients), drug-induced glaucoma (2 patients), infantile or juvenile glaucoma (2 patients), pigmentary glaucoma (2 patients), acute angle closure glaucoma (1 patient), and aphakic open angle glaucoma (1 patient).

^b^ Miscellaneous inactive choroidal neovascular membrane includes idiopathic (7 patients), degenerative myopia (3 patients), angioid streaks (1 patient), and ocular histoplasmosis (1 patient).

^c^ Other retina includes choroidal nevus (8 patients), cystoid macular edema (8 patients), degenerative myopia (8 patients), lattice degeneration (8 patients), sickle cell retinopathy (9 patients), macular dystrophy (7 patients), retinitis pigmentosa (7 patients), vitreomacular traction or adhesion (7 patients), retinoschisis (5 patients), hypertensive retinopathy (4 patients), juxtafoveal telangiectasia (4 patients), choroidal or retinal lesion (3 patients), cone-rod dystrophy (3 patients), full-thickness macular hole after repair (2 patients), ocular toxoplasmosis (2 patients), posterior vitreous detachmentor vitreous floaters (2 patients), proliferative diabetic retinopathy with history of tractional retinal detachment repair (2 patients), wet age-related macular degeneration declining treatment (2 patients), asteroid hyalosis (1 patient), disseminated chorioretinitis (1 patient), exudative retinopathy (1 patient), familial dominant drusen (1 patient), familial exudative vitreoretinopathy (1 patient), macular scar (1 patient), melanocytoma of optic nerve head (1 patient), optic disc drusen (1 patient), pigment epithelial detachment (1 patient), retinal pigment epithelial mottling of the macula (1 patient), and toxic maculopathy (1 patient).

^d^ Miscellaneous includes idiopathic intracranial hypertension (3 patients), keratoconus (2 patients), neurofibromatosis (2 patients), optic nerve edema (2 patients), optic neuritis (1 patient), optic neuropathy (2 patients), homonymous hemianopsia (1 patient), myopia with astigmatism (1 patient), pituitary mass (1 patient), retained lens following cataract surgery (1 patient), and schwannoma (1 patient).

Clinicians recommended patients from 2.7% of hybrid visits (25 visits) for a procedure visit such as an anti–vascular endothelial growth factor injection, anterior segment laser, or retinal laser. Additionally, 2.3% of hybrid visits (22 visits) led to a change in medication such as intraocular pressure–lowering agents, medical management of worsening macular edema, and idiopathic intracranial hypertension. Clinicians referred patients from 4.7% of all visits (44 visits) to see one of their colleagues for nonurgent consultation. The most common referrals were to the optometry service for refraction (10 referrals) or low-vision evaluation (3 referrals), and to the comprehensive service for cataract (10 referrals) or glaucoma (10 referrals) evaluation. There were no urgent referrals to another subspecialty for consultation. Details of the outcome measures are listed in Table 3.

**Table 3.** Visit Outcomes for All Completed Hybrid Visits in the Year 2020

Outcomes measures	Visits, No. (%) (N = 940)
Procedure visit	25 (2.7)
Intravitreal injection	15 (1.5)
Panretinal photocoagulation	2 (0.2)
Laser retinopexy	2 (0.2)
Photodynamic therapy	1 (0.1)
Nd:YAG capsulotomy	1 (0.1)
Selective laser trabeculoplasty	4 (0.4)
Medication adjustment	22 (2.3)
Addition of intraocular pressure–lowering agent	19 (2.0)
Medical management of macular edema	2 (0.2)
Restarted treatment for symptomatic idiopathic intracranial hypertension	1 (0.1)
Nonurgent referral	44 (4.7)
Optometry	
Refraction	10 (1.1)
Low vision	3 (0.3)
Comprehensive	
Cataract evaluation	10 (1.1)
Nd:YAG capsulotomy evaluation	3 (0.3)
Glaucoma evaluation	10 (1.1)
Neuro-ophthalmology evaluation	2 (0.2)
Cornea evaluation	1 (0.1)
Oculoplastic	1 (0.1)
Retina, new diabetic macular edema evaluation	1 (0.1)
Medical genetics	3 (0.3)
Urgent referral	0

Abbreviation: Nd:YAG, Neodymium-doped Yttrium Aluminum Garnet laser.

### Adverse Outcomes

Of the 940 completed hybrid visits, 671 (71.3%) returned to the eye clinic within 1 year. Only 16 patients (1.7%) required an additional standard in-person visit, either urgently during the imaging appointment or following the virtual appointment, to reach a management plan. No instances of irreversible vision loss were detected at these follow-up visits. Four cases were flagged by an ophthalmic technician for a same-day emergency evaluation by the on-call team. These were all patients with retinal disease with decreased vision for 3 patients and elevated intraocular pressure for 1 patient. These patients were subsequently referred to the retina clinic between 3 days to 6 weeks for follow-up and treatment without additional worsening of their vision and improved intraocular pressure after initiation of eye drops (Table 4).

**Table 4.** Imaging Appointments That Were Converted to a Same-Day, Emergency In-Person Visit With the On-Call Ophthalmology and Patients Who Were Brought Back for an In-Person Visit by the Same Clinician for Further Evaluation

Appointment No.	Service	Chief concern	Technician concerns	Findings	Outcome
Same-day emergency visits					
1	Retina	Dry AMD	Decreased VA	OCT macula with conversion to wet AMD	Retina visit in 1 week for anti-VEGF
2	Retina	NPDR	Elevated IOP	No neovascularization of the iris/angle, started on IOP drops	IOP normalized at 6-week follow-up
3	Retina	PDR	Decreased VA	New VH	Retina visit in 3 d, found to have TRD
4	Retina	TRD	Decreased VA	New VH	Retina visit in 2 weeks for anti-VEGF
Nonurgent in-person evaluation					
5	Retina	Lattice	NA	Referred for retinal hole, inadequate fundus imaging	Confirmed retinal hole, underwent laser retinopexy
6	Retina	NPDR	NA	Decreased VA, inadequate fundus imaging	New VH and cataract, scheduled for surgery
7	Retina	NPDR	NA	Decreased VA, inadequate fundus imaging	Conversion to PDR, underwent anti-VEGF injection
8	Retina	PDR	NA	Decreased VA, inadequate fundus imaging	No showed, seen 1 y later and diagnosed with macular ischemia
9	Retina	Vitreomacular traction	NA	New macular hole on OCT macula	Confirmed, scheduled for surgery
10	Retina	Macular hole	NA	Worsening macular hole on OCT macula	Confirmed, scheduled for surgery
11	Retina	NPDR	NA	Decreased VA with stable imaging	VH confirmed, underwent anti-VEGF
12	Retina	PDR	NA	Decreased VA with stable imaging	VA back to baseline
13	Retina	PDR	NA	IOP elevated, concern for neovascular glaucoma	Not detected, referred to glaucoma service
14	Retina	Retinitis pigmentosa	NA	Fundus imaging concerning for mass	No showed, patient died from metastatic lung cancer
15	Optometry	Glaucoma suspect	NA	Reporting eyelid pain and “bump”	Concern for trochleitis
16	Comprehensive	Primary open-angle glaucoma, scleritis	NA	Reported red eye	No evidence of scleritis

Abbreviations: AMD, age-related macular degeneration; IOP, intraocular pressure; NA, not applicable; NPDR, nonproliferative diabetic retinopathy; OCT, optical coherence tomography; PDR, proliferative diabetic retinopathy; TRD, tractional retinal detachment; VA, visual acuity; VEGF, vascular endothelial growth factor; VH, vitreous hemorrhage.

After completion of their hybrid visit, 12 patients were asked to return to the clinic for a standard in-person visit for further evaluation with the same clinician. These patients needed the additional evaluation because of inadequate imaging, new pathology requiring surgery, stable imaging with unknown vision loss, or new diagnosis requiring in-person evaluation. These extraneous in-person visits occurred a mean (SD) of 15.7 (12.0) days (range, 6-43 days) following the virtual appointment with the clinician, and the vast majority (10 visits) of these visits were performed by retina clinicians. Overall, our clinicians felt the hybrid visit model was able to provide adequate management 98.3% of the time. (Table 4).

## Discussion

The hybrid ophthalmology telemedicine model was envisioned during the COVID-19 pandemic to serve a population of patients with a history of stable, non–vision-threatening, non–procedure-requiring eye diseases, whose care was deemed nonurgent and at risk of being continually postponed. Within this patient population, we found in this cross-sectional study that the hybrid visit model was able to deliver quality care, particularly for glaucoma and retinal diseases.

### Importance of Patient Selection

The success of a hybrid visit starts at the very beginning with patient selection. Although clinicians were not restricted in how they used the hybrid model, clinicians were advised that hybrid visits were most suitable for stable, nonprocedural, non–vision-threatening diseases that could be evaluated with a standard protocol-based set of imaging or testing. For retinal diseases, it is important that the pathology could be reliably captured by ultrawidefield photography and adequately evaluated without stereopsis, so peripheral diseases, such as lattice degeneration or retinal holes, are less optimal than posterior diseases, such as macular degeneration. Patients must also have a certain baseline cognitive ability to interact and express themselves during the virtual appointment.

Although the hybrid ophthalmology telemedicine model was available to all ophthalmologists and optometrists in our eye clinic, it was up to the discretion of each clinician on how to use the model for their patients. A review of our data shows that, by far, clinicians used the hybrid visit for patients with glaucoma and retina-based diagnoses. This contrasts with studies on real-time, virtual-only ophthalmic visits. In Yale New Haven Hospital, the primary diagnoses for their virtual-only ophthalmic telemedicine program were oculoplastics and comprehensive ophthalmology.^12^ In a survey^13^ of Michigan Blue Cross Blue Shield’s billing data, cornea and external diseases accounted for 48.0% of all telemedicine visits, with retinal and glaucoma conditions accounting for only 16.8% and 13.4%, respectively. Other virtual-only ophthalmic telemedicine data^4,14,15^ have suggested a preference for oculoplastic, strabismus, neuro-ophthalmologic, and external surface diseases. The particular reliance on the evaluation of intraocular structures in glaucoma and retinal diseases, which cannot be captured during a telephone or video-based visit, and advances in both nonmydriatic ultrawidefield fundus photography and optical coherence tomography may explain why clinicians prefer to use hybrid visits for glaucoma and retinal diseases and reserve virtual-only visits for oculoplastics, strabismus, neuro-ophthalmic, and cornea and external diseases.

This suggestion that telemedicine models should be fitted to the disease can also be seen in other parts of medicine. Specialties that rely heavily on physical examinations capable of being emulated by images, such as dermatology, have reported particularly success with hybrid telemedicine systems,^16^ while specialties that rely less on physical examinations, such as psychiatry, may do better with virtual-only platforms.^17^

### Visit Adherence

During scheduling, patients are advised that both the imaging and virtual appointments are crucial components to a successful hybrid visit as issues arise if a patient missed either appointment. The equivalent scenario in a standard in-person visit would be if the patient did not receive a technician workup before seeing the clinician, or the patient left their appointment after a technician workup without seeing their clinician. Additionally, we were concerned that the need for 2 appointments would lead to lower visit adherence; however, our study found that the visit adherence for hybrid visits was actually better than the overall visit adherence for all eye clinic visits at the same time.

As the premise of the hybrid visit model is to emulate a standard in-person visit, ensuring the 2 appointments occur within a tight period was important for the ocular vitals and testing obtained during the imaging appointment to reflect the patient’s status during the virtual appointment accurately. We found that the majority (77.4%) of all completed hybrid visits had their virtual appointments completed within our protocol goal of 14 days after the imaging appointment.

### Adverse Outcomes

We did not identify any instances of irreversible vision loss due to a delay or misdiagnosis from a hybrid visit. Only 16 patients (1.7%) required an additional standard in-person visit, either urgently during the imaging appointment or following the virtual appointment, to reach a management plan. Therefore, our clinicians felt the hybrid visit model was able to provide adequate management 98.3% of the time. In fact, 5.5% of patients (49 patients) who had a hybrid visit in the calendar year 2020 had 2 visits with 1 patient reaching 3 visits. This high rate of success coupled with the clinical capacity limitations and desire by both patients and clinicians to reduce physical time in clinic reinforces the utility of hybrid visits for a carefully selected patient population.

### Limitations

This study was limited by its retrospective design using data from a single hospital-based eye clinic in the urban Northeast; therefore, generalizability to different health care systems may be limited. As telemedicine develops, we encourage other health care systems to review their own outcomes to ensure quality care.

## Conclusions

Hybrid visits are not intended to replace in-person visits. However, our study shows that overall, the hybrid ophthalmology telemedicine model was able to deliver quality care for a wide range of nonurgent, non–vision-threatening patient diseases, particularly in glaucoma and retina, and can serve as an alternative to the standard of care in-person visits when faced with space and safety limitations during a pandemic. It does not eliminate physical contact, but it does eliminate the need for dilation for patients with retinal diseases, leading to generally shorter appointment times and circumvents clinical capacity limitations. Novel therapeutics, vaccines, and the natural progression of the COVID-19 pandemic have made seeking in-person medical care safer than in the early days of the pandemic; nevertheless, the hybrid ophthalmology telemedicine model may serve as an alternative option to standard in-person visits for certain eye clinics with physical space and staffing limitations due to nonpandemic reasons or be applicable in the unfortunate event of a future pandemic.

## Notes

Supplement. **eTable 1. **Comparison Between the Standard of Care In-Person Visit With Different Forms of Telemedicine **eTable 2. **Imaging Protocols by Chief Concern
