# Supplementary material for: Outcomes of a Hybrid Ophthalmology Telemedicine Model for Outpatient Eye Care During COVID-19
Source: JAMA Netw Open. 2022 Aug 25;5(8):e2226292. doi: 10.1001/jamanetworkopen.2022.26292 (PMC9412225; doi:10.1001/jamanetworkopen.2022.26292)
Supplement: Supplement. — eTable 1. Comparison Between the Standard of Care In-Person Visit With Different Forms of Telemedicine eTable 2. Imaging Protocols by Chief Concern [file jamanetwopen-e2226292-s001.pdf]

Supplemental Online Content

Sanayei N, Albrecht MM, Martin DC, et al. Outcomes of a hybrid ophthalmology telemedicine model for outpatient eye care during COVID-19. *JAMA Netw Open*. 2022;5(8):e2226292. doi:10.1001/jamanetworkopen.2022.26292

eTable 1. Comparison Between the Standard of Care In-Person Visit With Different Forms of Telemedicine
eTable 2. Imaging Protocols by Chief Concern

This supplemental material has been provided by the authors to give readers additional information about their work.

© 2022 Sanayei N et al. *JAMA Network Open.*

eTable 1. Comparison between the standard of care in-person visit with different forms of telemedicine. The hybrid model can mimic all aspects of an in-person visit unlike other telemedicine models.

	Standard in-Person	Hybrid	Telephone	Video	Store-and-Forward
**Medical History**	✓	✓	✓	✓	
**Ocular Vitals**	✓	Limited			Limited
**Physical Examination**	✓	Proxy		Proxy	Proxy
**Testing**	✓	✓			✓
**Management**	✓	✓	✓	✓	✓

© 2022 Sanayei N et al. *JAMA Network Open.*

eTable 2. Imaging protocols by chief complaint

Hybrid Imaging Protocols	
Glaucoma Suspect (Glaucoma, Comprehensive, Optometry Services)	1. VA with and without pinhole
	2. Automated refraction
	3. IOP by two different methods (applanation and tonopen)
	4. HVF 24-2
	5. OCT RNFL
Retina	1. VA with and without pinhole
	2. IOP by tonopen
	3. Ultrawidefield fundus photographs
	4. Ultrawidefield fundus autofluorescence
	5. OCT macula
Keratoconus (Cornea Service)	1. VA with and without pinhole
	2. Automated refraction
	3. Automated keratometry
	4. Topography
Neuro-ophthalmology	1. VA with and without pinhole
	2. IOP
	3. Color plates
	4. OCT RNFL/GCL
	5. Optic nerve photographs

GCL: ganglion cell layer, HVF: Humphrey visual field, IOP: intraocular pressure, OCT: optical coherence tomography, RNFL: retinal nerve fiber layer, VA: visual acuity

© 2022 Sanayei N et al. *JAMA Network Open.*
